# Mouse Mesenchymal Progenitor Cells Expressing Adipogenic and Osteogenic Transcription Factors Suppress the Macrophage Inflammatory Response

**DOI:** 10.1155/2017/5846257

**Published:** 2017-01-16

**Authors:** Natalie Fernandez, Heather Renna, Lauren McHugh, Katie Mazolkova, William Crugnola, Jodi F. Evans

**Affiliations:** ^1^Department of Biology, Chemistry and Environmental Studies, Molloy College, 1000 Hempstead Avenue, Rockville Centre, NY 11570, USA; ^2^Biomedical Research Core, Winthrop University Hospital, 101 Mineola Blvd., Mineola, NY 11501, USA; ^3^Stony Brook University School of Medicine, Stony Brook, NY 11794, USA

## Abstract

Mesenchymal progenitor cell characteristics that can identify progenitor populations with specific functions in immunity are actively being investigated. Progenitors from bone marrow and adipose tissue regulate the macrophage (MΦ) inflammatory response by promoting the switch from an inflammatory to an anti-inflammatory phenotype. Conversely, mesenchymal progenitors from the mouse aorta (mAo) support and contribute to the MΦ response under inflammatory conditions. We used cell lines with purported opposing immune-regulatory function, a bone marrow derived mesenchymal progenitor cell line (D1) and a mouse aorta derived mesenchymal progenitor cell line (mAo). Their interaction and regulation of the MΦ cell response to the inflammatory mediator, lipopolysaccharide (LPS), was examined by coculture. As expected, D1 cells suppressed NO, TNF-*α*, and IL-12p70 production but MΦ phagocytic activity remained unchanged. The mAo cells enhanced NO and TNF-*α* production in coculture and enhanced MΦ phagocytic activity. Using flow cytometry and PCR array, we then sought to identify sets of MSC-associated genes and markers that are expressed by these progenitor populations. We have determined that immune-supportive mesenchymal progenitors highly express chondrogenic and tenogenic transcription factors while immunosuppressive mesenchymal progenitors highly express adipogenic and osteogenic transcription factors. These data will be useful for the isolation, purification, and modification of mesenchymal progenitors to be used in the treatment of inflammatory diseases.

## 1. Introduction

Multipotent stromal cells also known as mesenchymal stem cells (MSC) are nonhematopoietic stem/progenitor cells originally isolated from the bone marrow that give rise to connective tissues in the embryo and are present in adult mammalian tissues [1]. MSC are active in the repair of tissues by replacing damaged cells but more recently they have been studied for their immunoregulatory properties [1, 2]. Due to their capacity to suppress and regulate inflammation through direct and indirect contact with T-cells and macrophage cells, MSC are ideal candidates for use in cell-based therapies of inflammatory diseases. Phase II and phase III clinical trials investigating the therapeutic use of MSC in treating graft-versus-host disease, Crohn's disease, progressive multiple sclerosis, kidney transplant rejection, and ischemic cardiomyopathy are in progress [3].

MSC suppress T-cell responses by reducing their proliferation [4, 5], inducing apoptosis [6], and supporting the differentiation of the regulatory T-cell phenotype [7]. MSC regulation of macrophage cells (MΦ) has not been as extensively studied and current studies have disparate results. MSC derived from bone marrow, adipose, and placental tissues promote the differentiation of the anti-inflammatory macrophage phenotype [8–10]. On the other hand, macrophage-associated MSC can also display an inflammatory phenotype [11] and recent work has revealed that mesenchymal progenitor cells from the mouse aorta (mAo) contribute to and support the inflammatory macrophage [12]. The MSC phenotypic characteristics that underlie their anti-inflammatory properties are under active investigation and many recent studies explore the relationship between heterogeneity in surface antigen expression and heterogeneity in functional properties among MSC [13].

In this study, we compared the ability of the mAo mesenchymal progenitor and a bone marrow derived mesenchymal progenitor cell line (D1) to regulate the inflammatory response of MΦ. mAo and D1 cells were cocultured with bone marrow derived MΦ and exposed to lipopolysaccharide (LPS). Nitric oxide (NO), tumor-necrosis factor-*α* (TNF-*α*), and interleukin-12 (IL-12) were used as markers of inflammation. Additionally, we examined the effect of coculture on the phagocytic index of MΦ in response to zymosan exposure. Then, to correlate their immune-regulatory capacity to their phenotypic profile, we measured the MSC surface antigens CD44, CD73, Sca-1, CD105, and CD106 using flow cytometry and measured expression of MSC-associated genes using a PrimePCR mesenchymal stem cells PCR gene array.

## 2. Materials and Methods

### 2.1. Materials

All cell culture media, trypsin, FBS, and antibiotic/antimycotic solutions were obtained from Invitrogen (Carlsbad, CA). The L-929 fibroblast (CCl-1) was purchased from the American Type Tissue Collection (Manassas, VA). The FITC rat anti-mouse CD44 (Cat. #553133), PE rat anti-mouse CD 105 (Cat. # 562759), PE rat anti-mouse Ly-6AE (Sca-1) (Cat. # 561076), FITC rat anti-mouse CD45 (Cat. #553080), FITC rat anti-mouse CD106 (Cat. #553332), PE rat anti-mouse CD73 (Cat. #557041), and FITC rat anti-mouse CD11b (Cat. #553310) were purchased from BD Biosciences. Gamma irradiated LPS from* E. coli* (#L4391) and all other chemicals and reagents were purchased from Sigma-Aldrich (USA) unless otherwise specified.

### 2.2. Animals

All animal protocols were approved by the Winthrop University Hospital's Animal Care and Use Committee and adhere to the regulations outlined by the National Institutes of Health. C57BL/6 male mice were obtained from Taconic, North America. Animals were housed under local vivarium conditions (12 h light-dark cycle) and allowed to acclimate for at least 7 days prior to experimentation. Mice were euthanized under CO_2_ at 8–12 weeks of age and aorta and hind limbs were removed in preparation for cell isolation.

### 2.3. Cell Culture

#### 2.3.1. Aortic Mesenchymal Progenitor Cell Line (mAo)

Mouse aortic progenitor lines were derived from C57BL/6 mice using the method of da Silva Mierelles [14] with slight modification as described in [15].

#### 2.3.2. Bone Marrow Derived Mesenchymal Progenitor Cells

(D1) D1 ORL UVA (D1) cells, a bone marrow-derived mesenchymal stem cell line, were obtained from the American Type Culture Collection (ATCC #CRL-12424) and maintained in DMEM supplemented with 10% FBS, 100 U/mL penicillin sodium, 100 U/mL streptomycin sulfate, and 0.25 *µ*g/mL amphotericin B.

#### 2.3.3. Bone Marrow-Derived Macrophage Cells (MΦ)

Bone marrow from the hind limbs of the C57BL/6 mouse was isolated as previously described [16]. After creating a single-cell suspension, nucleated cells were counted using 3% acetic acid/trypan blue exclusion and plated in DMEM supplemented with 10% fetal bovine serum (FBS), 15% L929 fibroblast cell conditioned medium, 100 U/mL penicillin sodium, 100 U/mL streptomycin sulfate, and 0.25 *µ*g/mL amphotericin B at 10^7^ cells per 100 mm Petri dish. The L929 conditioned medium was prepared as suggested by the ATCC. The L-929 cell line produces M-CSF which supports the growth and differentiation of macrophages from the bone marrow. After 3 days, half the medium was removed and replaced with fresh medium. A complete medium change was performed on day 6. At day 7 of culture monocyte/macrophage cells were replated according to experimental objectives, passed or frozen, and stored in liquid nitrogen (LN_2_). Cultures up to passage 2 were used in experiments.

#### 2.3.4. Coculture

The mAo cells were initiated at a density of 1.5 × 10^4^/cm^2^ and the D1 were initiated at a density of 3.25 × 10^3^/cm^2^. The cells were initiated at different densities because when plated at the same density, the D1 cells will reach confluence earlier. In order to carry out the coculture experiments with both cell populations with the same timeline, it was necessary to initiate the D1 cells at a lower density. They have similar cell numbers at confluence. When the mesenchymal progenitor cultures reached confluence, MΦ were added at a density of 1.0 × 10^5^/cm^2^ and allowed to attach overnight. Cultures were then washed with serum-free DMEM supplemented with 0.02% BSA and then left untreated or were treated with LPS (100 ng/ml) for 24 h in fresh serum-free DMEM/0.02% BSA. Culture supernatants were then collected and stored at −80°C until assay.

### 2.4. Flow Cytometry

For staining of mAo MSC and D1 MSC surface markers, single-cell suspensions were first incubated with Fc receptor blocking reagent (Miltenyi Biotec) followed by staining with antibodies against surface markers CD29, CD44, CD73, CD105, CD106, Sca-1 (Ly-6AE), and CD45 for 30 min at 4°C. After incubation, cells were washed twice in wash buffer (1% FBS in PBS) and analyzed with an Accuri C6 flow cytometer.

### 2.5. Nitrite Measurements

Nitrite, as a reflection of nitric oxide (NO) production, was measured in cell culture supernatant using the Griess Reagent system (Promega, Madison, WI) according to the manufacturer's instructions.

### 2.6. Secreted Cytokine and Chemokine Measurements

TNF-*α* and IL-12p70 cytokines were measured in culture supernatants using Ready-SET-Go! ELISA kits from eBioscience (San Diego, CA).

### 2.7. Phagocytosis Assay

MΦ and cocultures of MΦ/mAo and MΦ/D1 were initiated in 4-well chamber slides and incubated overnight. Zymosan-A* S. cerevisiae* BioParticles®, fluorescein conjugate (Molecular Probes), was added at 5 and 10 particles per MΦ cell and incubated for 45 minutes. The chambers were then rinsed gently 4x with cold PBS and fixed in 4% paraformaldehyde (PFA) followed by mounting in hard-set DAPI mounting medium (Vector Laboratories) to stain the nuclei. Ten to twelve fluorescent digital micrographs were taken of each culture type and analyzed for intracellular zymosan-A. Phagocytic cells were categorized into groups according to the number of ingested particles and data is presented as [1–5], [6–10], [11–15], and [16+] particles per cell.

### 2.8. Gene Expression

#### 2.8.1. Gene Array

A PrimePCR mesenchymal stem cells (SAB target list) PCR gene array from Bio-Rad was used to compare the MSC gene-expression profiles of the mAo and D1 cultures. RNA was extracted from the cultures using TRIzol reagent (Invitrogen) according to the manufacturer's instructions. After quantization, 2 *µ*g total RNA was reversed transcribed using the iScript Advanced cDNA Synthesis kit (Bio-Rad). The reaction mix was prepared as directed using Sso Advanced Universal SYBR Green supermix (Bio-Rad) to yield 20 ng cDNA per reaction in the PCR array.

#### 2.8.2. Real-Time Reverse Transcription PCR

Real-time reverse transcription PCR was carried out using Sso Advanced Universal SYBR Green supermix and the CFX96 Touch Real-Time PCR System from Bio-Rad. Primer sequences were specific for mouse: Pparg forward 5′-CGGGCTGAGAAGTCACGTT-3′ Pparg reverse 5′–TGTGTCAACCATGGTAATTTCAGT-3′, Sox2 forward 5′–GATCAGCATGTACCTCCCCG-3′ Sox2 reverse 5′–TCCTCTTTTTGCACCCCTCC-3′, Sox9 forward 5′-GGGCGAGCACTCTGGGCAAT-3′ Sox9 reverse 5′-CGTCGCGGAAGTCGATGGGG-3′, and Runx2 forward 5′- CCCTGAACTCTGCACCAAGT-3′ Runx2 reverse 5′-TGGAGTGGATGGATGGGGAT-3′. PCR conditions were 95°C for 5 min followed by 40 cycles of 95°C 10 s, 55°C, 10 s, and 72°C for 30 s.

### 2.9. Statistical Analyses

Unless otherwise indicated, data were analyzed using one-way ANOVA. Bonferroni's posttest was used to determine statistical differences between groups.

## 3. Results

### 3.1. When in Coculture with MΦ, mAo MSC Enhance While D1 MSC Suppress LPS-Induced Inflammation

Nitric oxide (NO) is a hallmark of inflammation and is produced by MΦ in an acute response to inflammatory stimuli [17] but can also be produced by MSC to regulate T-cells [2, 18]. Previous studies have indicated that mAo progenitors produce NO synergistically in coculture with MΦ [12]. Although many previous studies have determined that bone marrow derived progenitors suppress MΦ secretion of inflammatory cytokines, there is a paucity of studies that have examined the production of NO in cocultures of bone marrow derived progenitors and MΦ. Here we compared the production of NO in D1/MΦ with that of mAo/MΦ cocultures after exposure to LPS.

**Figure 1:**
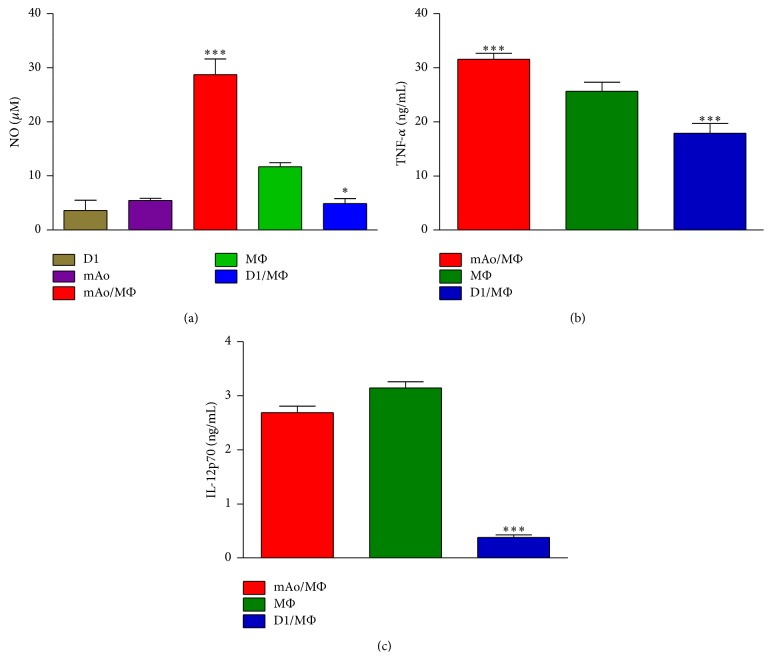
When in coculture with MΦ, aortic mesenchymal progenitor cells, mAo, enhance while bone marrow-derived mesenchymal progenitor cells, D1, suppress LPS-induced NO, TNF-*α*, and IL-12p70 production. Nitrite production as a measure of NO (a), TNF-*α* (b), and IL-12p70 (c) was measured in culture supernatants of mAo MSC, D1 MSC, and MΦ cultured alone and in coculture. Cultures were treated with LPS (100 ng/mL) for 17 hr. Data are presented as mean ± SEM and are representative of 3 experiments each with *n* = 4. ^**∗**^Significantly different from MΦ alone, *P* < 0.05, ^*∗∗∗*^significantly different from MΦ alone, *P* < 0.0001.

As previously determined [12], the mAo/MΦ culture produced NO at synergistic levels significantly above MΦ or mAo cells cultured alone (~28 *μ*M in mAo MSC/MΦ versus ~11 *µ*M in MΦ and ~5 *µ*M in mAo MSC, *P* < 0.001) (Figure 1(a)). In T-cell studies, NO produced by MSC results in immunosuppression of T-cells in close proximity [19]. MΦ also use NO to mediate their cytotoxic effects but are also susceptible to apoptosis upon exposure to NO. Whether in the case of mAo/MΦ interaction enhanced NO production leads to MΦ apoptosis and the subsequent suppression of the innate immune response and/or contributes to the suppression of T-cell responses remains to be determined. In contrast, the D1/MΦ coculture produced significantly less NO when compared to MΦ cultures (~5 *µ*M in D1/MΦ versus ~11 *µ*M in MΦ, *P* < 0.05) (Figure 1(a)). The significant decrease in NO production in the D1/MΦ cultures is in line with previous studies demonstrating that bone marrow mesenchymal progenitors promote the switch of the MΦ from the inflammatory to anti-inflammatory phenotype [8, 9].

IL-12p70 and TNF-*α* are not expressed by mesenchymal progenitors [12, 20, 21] and therefore these cytokines were used to examine the inflammatory profile of MΦ while in coculture with mAo and D1 progenitor cells. Cytokine secretion of the cocultured cells was compared to that of the MΦ cells cultured alone. Coculture with mAo increased TNF-*α* secretion by MΦ (~26 ng/mL in MΦ versus ~32 ng/mL in mAo MSC/MΦ, *P* < 0.001) and in D1/MΦ cocultures it was significantly reduced (~18 ng/mL in D1/MΦ versus ~26 ng/mL in MΦ, *P* < 0.001) (Figure 1(b)). IL-12p70 secretion in mAo/MΦ cultures did not significantly deviate from MΦ cultures (~2.8 ng/mL versus ~3.2 ng/mL, resp.) (Figure 1(c)). On the other hand, in D1/MΦ cultures, IL-12 secretion was significantly reduced (~3.2 ng/mL in MΦ versus ~0.4 ng/ml in D1/MΦ, *P* < 0.001) (Figure 1(c)). These data establish that the D1 bone marrow derived progenitors suppress while those from the aorta (mAo) contribute to the inflammatory profile of MΦ activated by LPS consistent with previous works [10, 22].

### 3.2. The Phagocytic Index of MΦ Is Increased in Response to Zymosan When in Coculture with mAo

**Figure 2:**
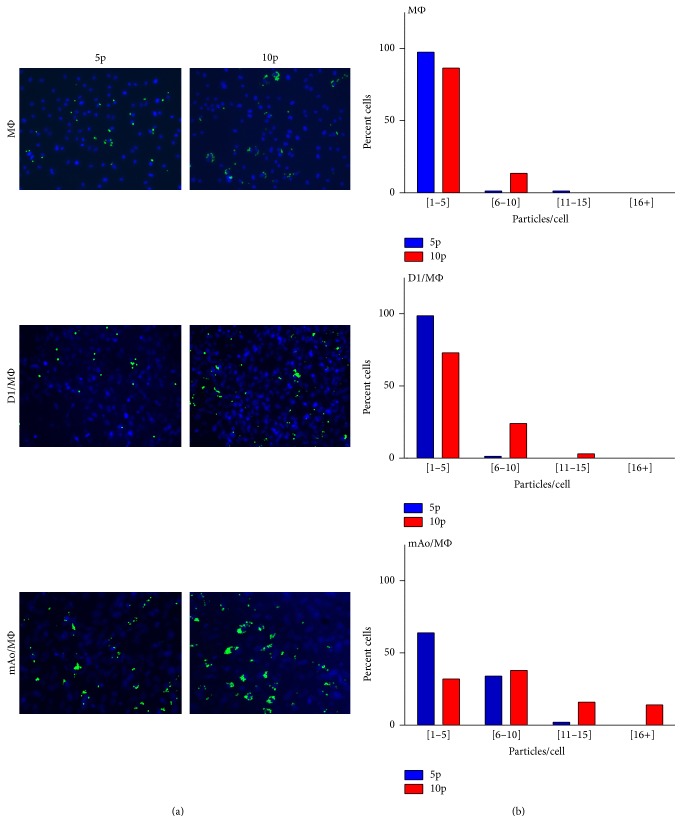
The phagocytic index of MΦ is increased when in coculture with aortic mesenchymal progenitor cells, mAo. Fluorescent micrographs of MΦ, mAo/MΦ, and D1/MΦ cultures exposed to 5 and 10 particles of FITC labeled zymosan-A per MΦ cell. Cultures were counterstained with DAPI to delineate nuclei (a). Micrographs were used to quantify MΦ uptake of zymosan particles. Counts per cell are presented in the following ranges: [1–5], [6–10], [11–15], and [16+] zymosan-A particles per cell (b). Data represent findings from 3 experiments, each with an *n* = 4.

In addition to establishing the impact of MΦ and mesenchymal progenitor cell-cell interaction on the inflammatory milieu, we also examined the impact on MΦ phagocytic activity. MSC are known to induce an increase in MΦ phagocytosis consistent with their promotion of the anti-inflammatory phenotype [23]; however, the influence the mAo and D1 cells have on MΦ phagocytosis has not been previously determined. Zymosan-A particles were added to cocultures of D1/MΦ and mAo/MΦ at a density of 5 and 10 particles per MΦ cell, levels below saturation as determined in preliminary studies. Ingested, cell-associated zymosan-A particles were counted and results were presented as ranges representing different levels of activity. The ranges of increasing activity include [1–5], [6–10], [11–15], and [16+] particles ingested per cell (Figures 2(a) and 2(b)). A shift to the right in the bar graph (from the low to high ranges) correlates with an increase in phagocytic activity.

Coculture with D1 had no effect on MΦ phagocytosis of zymosan-A particles at 5 or 10 particles per cell; note the similar pattern in bar graphs of D1/MΦ and MΦ in Figure 2(b). Additionally, when MΦ were in coculture with the mAo, the phagocytic activity of the MΦ was enhanced, reflected in a shift of the mAo/MΦ bar graph to the right when compared to MΦ in Figure 2(b). Taken together, data from the cytokine and phagocytosis assays indicate that D1 progenitors suppress LPS-induced inflammatory pathways but have no effect on those induced by zymosan. The mAo progenitors however support both LPS-induced and zymosan-induced MΦ activity.

### 3.3. Correlation of mAo and D1 Phenotypic Profiles with Their Immune-Regulatory Functions

After we established the immune-regulatory functions of mAo and D1 cells when in coculture with MΦ under inflammatory conditions, we then sought to determine their phenotypic and genotypic profiles. Our goal was to relate their functional properties to their expression of mesenchymal progenitor cell-associated markers.

#### 3.3.1. MSC-Associated Antigen Expression

**Figure 3:**
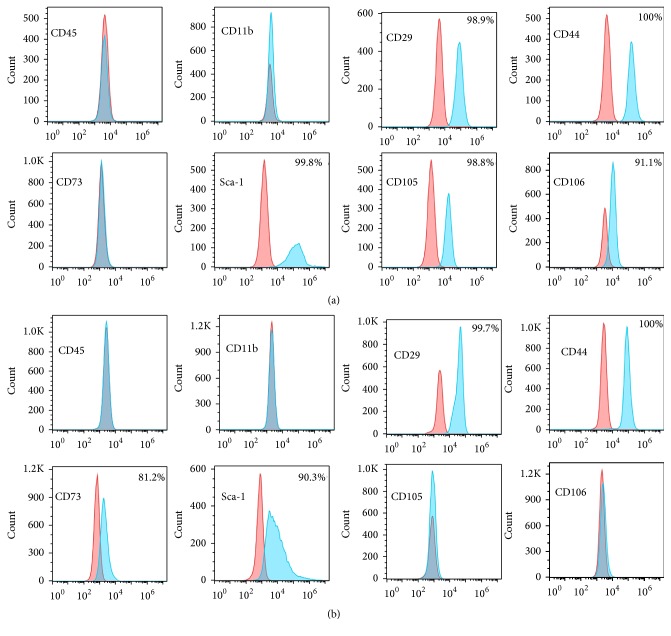
Flow cytometry of MSC-associated antigens in aortic mesenchymal progenitor cells, mAo, and bone marrow-derived mesenchymal progenitor cells, D1. Flow cytometry was performed to detect CD45, CD11b, CD44, CD73, Sca-1, CD105, and CD106 surface antigens in mAo (a) and D1 (b) cultures.

As expected, both cell populations are negative for the hematopoietic markers, CD45 and CD11b. Both cell types were also positive for the MSC-associated antigens, CD29, CD44, and Sca-1, but differed in their expression of CD73, CD105, and CD106. The D1 cell cultures are heterogeneous for the expression of the CD73 marker but negative for CD105 and CD106. On the other hand, mAo cells are negative for CD73 and positive for CD105 and CD106 (Figures 3(a) and 3(b)). Results of the MSC gene specific reverse transcription PCR array confirmed the differences in expression of CD73, CD105, and CD106 and also uncovered differential expression of CD90 (Table 1).

In 2006, the Mesenchymal and Tissue Stem Cell Committee of the International Society for Cellular Therapy published a position paper outlining minimum criteria for the MSC designation in human cell populations. The minimum criteria are that MSC must be capable of multipotent differentiation, be negative for leukocyte specific antigens, and at a minimum express the cell surface markers, CD73, CD90, and CD105 [24]. For mouse cells, however, no clear criteria have emerged and there are many differences in MSC surface antigen expression between mouse and human cells [25]. There is also a great variation in reported MSC surface antigen expression and many new potential identifying antigens are continually emerging [26]. Both the D1 and mAo cells used in these studies are capable of multipotent differentiation [22] but have differing surface antigen profiles and therefore we use the term mesenchymal progenitor.

Expression of CD73 on the D1 cells is in line with their downregulation of the inflammatory environment in the cocultures. CD73 is a 5′ ectonucleotidase, an enzyme that in tandem with CD39 is responsible for catalyzing the second step in the generation of adenosine from the adenosine triphosphate released from cells into the extracellular fluid. Adenosine then acts on adenosine receptors expressed on leukocytes and generally suppresses their inflammatory response [27]. CD73 expressed on MSC contributes to immunosuppression of T-cell activity during autoimmune responses [28]. However, because the D1 cell cultures are heterogeneous for CD73, direct evidence of CD73 in D1 mesenchymal progenitor regulation of MΦ is warranted. Moreover, due to their location, surface antigens are very likely involved in cell-cell regulatory interaction between the mesenchymal progenitors and MΦ. Therefore, the potential roles of CD105 and CD106 in the regulatory function of the mAo progenitors should also be a focus of future investigations.

#### 3.3.2. PCR Array Analyses

The PCR array used in our studies encompasses 92 genes associated with mesenchymal stem cells, including the surface marker genes discussed above. When focusing on the results of the array, we used 30 cycles as a stringent threshold Cq value to determine expression. We then categorized the expression differences as (1) differentially expressed, (2) equivalently expressed, or (3) expressed in neither cell population. Only genes with ≥3-fold expression difference were considered to be differentially regulated. The raw gene array data can be found in Supplemental File 1 (in Supplementary Material available online at https://doi.org/10.1155/2017/5846257). Genes that were equivalently expressed or not expressed in the two cell populations were discounted as being the reason for their opposing immune-regulatory functions. We then classified the differentially expressed genes according to function or utility. The classifications used were MSC-associated surface antigens, transcription factors, growth factors, matrix/cell adhesion/cytoskeletal elements, and immune-related/miscellaneous (Table 1).

#### 3.3.3. MSC-Associated Gene-Expression Patterns

**Figure 4:**
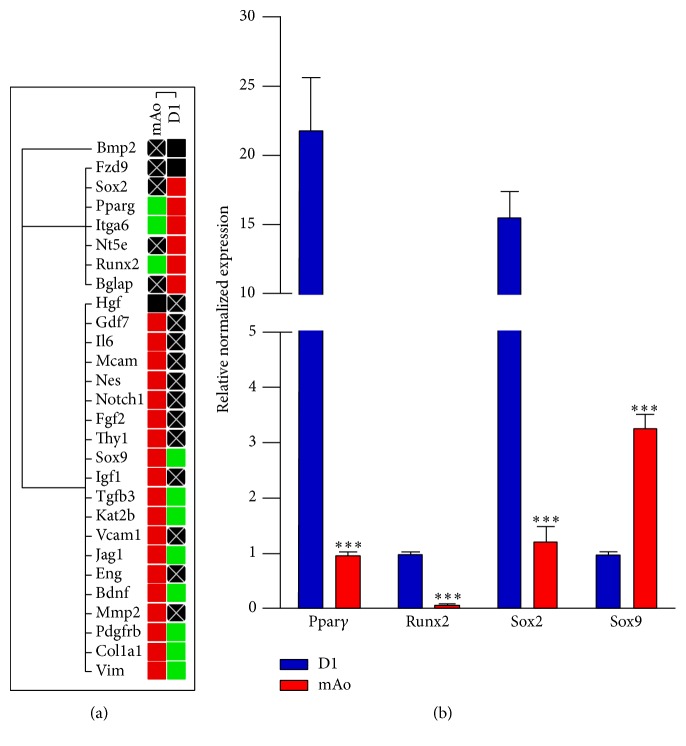
Mesenchymal stem cell-associated genes differentially expressed in mAo and D1 progenitor cells. (a) Clustergram demonstrating differentially expressed genes after normalizing to Gapdh, Hprt, and Tbp as shown in Table 1. Relative expression is indicated in the order of the target genes with the lowest expression at the top of each cluster. Red indicates greater expression, green indicates lower expression, black indicates very low expression, and black with a white X indicates no expression (Cq ≥ 30). (b) Relative expression of the Ppar*γ*, Runx2, Sox2, and Sox9 transcription factors in mAo and D1 progenitor cells using the Gapdh gene to normalize. Data are presented as mean ± SD, *n* = 3 separate experiments. Significant differences were determined using *t*-test. ^*∗∗∗*^*P* < 0.0001.

*Transcription Factors*. Patterns of transcription factor genes demonstrate that SRY-box containing gene 2 (Sox2) is expressed in D1 but not in mAo progenitors. Peroxisome proliferator activated receptor *γ* (Ppar*γ*) and runt related transcription factor 2 (Runx2) also exhibit a greater expression in D1 than in mAo by 8.12- and 7.26-fold, respectively. On the other hand, growth differentiation factor 7 (Gdf7/Bmp12), K(lysine) acetyltransferase 2B (Kat2B/PCAF), and SRY-box containing gene 9 (Sox9) are more highly expressed in mAo cells compared to D1 (Figure 4 and Table 1). Using real-time reverse transcription PCR, we were able to confirm the differential regulation of Sox2, Ppar*γ*, Runx2, and Sox9 among D1 and mAo mesenchymal progenitor populations (Figure 4(b)).

Ppar*γ* is a key regulator of adipocyte differentiation and glucose homeostasis [32]. Runx2 protein is essential for osteoblast differentiation and skeletal morphogenesis and acts as a scaffold for nucleic acids and regulatory factors involved in skeletal gene expression [33]. Sox2 is essential for self-renewal and proliferation of osteoblast precursors [34]. The fact that these transcription factors are all highly expressed in D1 cells indicates that they are poised for adipogenic and/or osteogenic differentiation. Gdf7 is a key regulator of tenogenic differentiation of mesenchymal stem cells [42]. Sox9 is essential for mesenchymal condensation prior to chondrogenesis and for inhibiting chondrogenic hypertrophy while Kat2B/PCAF is a chromatin histone acetyltransferase involved in transactivation of chondrogenic genes [25]. Upregulated expression in Gdf7, Kat2B/PCAF, and Sox9 suggests that mAo are primed for chondrogenic/tenogenic differentiation.

*Growth Factors*. The patterns of differentially expressed growth factor genes among D1 and mAo progenitor populations are in line with patterns of transcription factor expression (Figure 4(a) and Table 1). Bone gamma carboxyglutamate protein (Bglap) and bone morphogenetic protein 2 (Bmp2) are expressed in D1 cells and not in mAo cells. Bmp2 is an osteogenic marker and its expression induces osteoblast differentiation [39] while Bglap is a highly conserved protein that participates in ossification and is associated with a mineralized bone matrix [37, 38]. Platelet derived growth factor receptor, beta polypeptide (Pdgfrb) expression was 7.21-fold greater in mAo compared to D1 cells and fibroblast growth factor 2 (Fgf2) is only expressed in mAo cells. Pdgfb and Fgf2 both stimulate MSC proliferation and migration of MSC, while inhibiting their adipogenic and osteogenic differentiation [40, 41, 45]. Transforming growth factor beta 3 (Tgfb3), a growth factor that influences chondrogenic differentiation, is expressed 3.52-fold greater in mAo compared to D1 cells [46].

*Extracellular Matrix, Cell Adhesion, and Cytoskeletal Elements*. When examining expression of genes associated with the extracellular matrix, cell adhesion, and cytoskeletal elements (Figure 4(a) and Table 1), we found melanoma cell adhesion molecule (Mcam) expression was detectable in mAo progenitors but not in D1 cells consistent with the perivascular origin of the mAo [49, 50]. Matrix metallopeptidase 2 (Mmp2), nestin (Nes), and vascular cell adhesion molecule 1 (Vcam1) were also expressed in mAo but not in D1 cells. Collagen, type I, alpha 1 (CoI1*α*1), and Vimentin (Vim) expression were 3.03- and 5.05-fold, respectively, greater in mAo compared to D1 cells while Integrin alpha 6 (Itga6) expression was 3.25-fold greater in D1 cultures. Expression of Mmp2 and Vim in mAo provides a mechanism for their migration within the perivascular region [51, 55]. Vcam1 mediates the attachment of hematopoietic cells and serves as a marker for MSC with T-cell immunosuppressive activity [53, 54] pointing to mechanisms through which the mAo can interact with immune cells. In contrast, Itga6 expression is greater in D1 and functions to enhance MSC multipotency through specific transcription factors like Oct4 and Sox2 [48], while nestin, which is also associated with multipotency [52], is expressed only by the mAo cells. Therefore, mAo and D1 progenitor cells express genes which suggest they maintain their proliferative capacity and multipotency through different mechanisms.

*Immune-Related/Miscellaneous*. The genes that are related to immune function (Figure 4(a), Table 1) include jagged 1 (Jag1), interleukin-6 (IL-6), leukemia inhibitory factor (Lif), and notch gene homolog 1 (Drosophila) (Notch1). Notch1, only present in mAo cells, is a cell surface receptor for Jag1 which is expressed 22.11-fold greater in mAo versus D1 cells. The Notch1/Jag1 signaling pathway is required for MSC induction of regulatory T-cell expansion and points to the potential for mAo progenitor interaction and regulation of the adaptive immunity [59]. In line with this, Il-6, a cytokine that when secreted by MSC inhibits lymphocyte apoptosis [60] and suppresses dendritic cell differentiation [61], is expressed only by mAo progenitors. Lif transcripts were also only detected in mAo cells. The product of this gene is involved in upregulation of MSC pluripotency markers [62].

Frizzled homolog 9 (Fzd9) does not fit any category yet described and falls under miscellaneous. Fzd9 is expressed by D1 and not mAo cells (Figure 4(a), Table 1). This gene is upregulated during early osteogenic differentiation [58] and its expression by D1 cells is consistent with their osteogenic transcription and growth factor gene-expression profile.

## 4. Summary

Data from the cytokine phagocytosis analyses establish that the D1 bone marrow derived progenitors suppress the MΦ inflammatory profile but have no effect on zymosan-induced MΦ phagocytosis, while progenitors derived from the aorta (mAo) contribute to MΦ inflammation activated by LPS and support zymosan-induced MΦ activity.

The correlation of immune function and gene array studies revealed three major themes. (1) Expression patterns of transcription and growth factors suggest that the mAo mesenchymal progenitors, which enhance MΦ inflammatory responses, are poised to differentiate into chondrocytes, while D1 mesenchymal progenitors, which suppress MΦ inflammatory responses, are poised to differentiate into osteoblasts and adipocytes. (2) mAo mesenchymal progenitors express genes which are consistent with interaction and regulation of the adaptive immunity. (3) The transcriptional patterns of the D1 mesenchymal progenitor and the aortic tissue derived mAo mesenchymal progenitor suggest that they maintain their proliferative capacity and multipotency through different mechanisms. It must be noted that in addition to tissue source these progenitor populations differ in passage number, potentially contributing to the phenotypic and immunoregulatory differences observed in these studies.

## 5. Conclusion

These studies demonstrate a functional heterogeneity among mesenchymal progenitor populations derived from different tissues in the regulation of macrophage cells. A major finding indicates that mesenchymal progenitors expressing surface antigens, transcription, and growth factors associated with adipogenic and osteogenic differentiation suppress LPS-induced macrophage inflammation. These data will be useful for the isolation, purification, and modification of mesenchymal progenitors to be used in the treatment of inflammatory diseases.

## Supplementary Material

Supplementary File 1 is an excel file which includes the run file for the PrimePCR mesenchymal stem cells (SAB target list, Bio-Rad) gene array (sheet 1), the raw data with analysis notations (sheet 2), and a summary of expression differences (sheet 3).

## Figures and Tables

**Table 1:** Genes differentially expressed in mAo and D1 progenitor stem cells.

	Gene symbol	ΔCq mAo/D1	Specific function
*MSC-associated surface antigens*			
Endoglin (CD105)	Eng	(+/−)	Coreceptor for TGF*β*1 and TGF-*β*3 [29]. Expression on mouse MSC is heterogeneous and expression reduces adipogenic and osteogenic differentiation capacity and increases capacity for inhibition of T-cell proliferation [13].
5′-nucleotidase, ecto (CD73)	Nt5e	(−/+)	Generates extracellular adenosine by dephosphorylation of adenosine 5′-monophosphate and plays a role in MSC osteogenic differentiation [30].
Thymus cell antigen 1, theta (CD90)	Thy1	(+/−)	Originally discovered as a thymocyte antigen. Blocks adipogenesis and PPAR*γ* [31].
*Transcription factors*			
K(lysine) acetyltransferase 2B	Kat2b	4.27	Also known as P300/CBP-associated factor (PCAF). A chromatin histone acetyltranferase involved in transactivation of chondrogenic genes [25].
Peroxisome proliferator activated receptor gamma	Pparg	−8.12	Nuclear receptor and established major inducer of adipogenesis [32].
Runt related transcription factor 2	Runx2	−7.26	Also known as Cbfa1. A bone transcription factor involved in the osteogenic differentiation of MSC [33].
SRY-box containing gene 2	Sox2	(−/+)	An embryonic transcription factor that regulates lineage differentiation and proliferation of human MSC [34].
SRY-box containing gene 9	Sox9	3.17	A major chondrogenic transcription factor that is also involved in regulation of proliferation and adipogenic and osteogenic differentiation of MSC [35].
*Growth factors*			
Brain derived neurotrophic factor	Bdnf	4.93	Stimulates nerve cell differentiation and maturation; MSC secreted BDNF protects nerve cells from apoptosis and enhances endogenous neurogenesis [36].
Bone gamma carboxyglutamate protein	Bglap	(−/+)	Highly conserved protein associated with mineralized bone matrix [37]. Also known as osteocalcin; used as a marker of MSC osteogenic differentiation [38].
Bone morphogenetic protein 2	Bmp2	(−/+)	Facilitates the osteogenic differentiation of MSC [39].
Fibroblast growth factor 2	Fgf2	(+/−)	Promotes proliferation [40] and downregulates senescence in BMMSC cultures [41].
Growth differentiation factor 7	Gdf7	(+/−)	Promotes tenogenic differentitation of mesenchymal stem cells [42].
Hepatocyte growth factor	Hgf	(+/−)	HGF and its primary receptor cMET play a critical role in MSC stimulated recovery in experimental autoimmune encephalomyelitis [43].
Insulin-like growth factor 1	Igf1	(+/−)	Treatment of MSC with IGF-I increases their engraftment in a rat model of myocardial infarction [44].
Platelet derived growth factor receptor, beta polypeptide	Pdgfrb	7.21	A cell surface tyrosine kinase receptor for members of the platelet-derived growth factor (PDGF) family. Blocks adipogenic differentiation by blocking PPARy and CEPB*α* expression; promotes MSC self-renewal [45].
Transforming growth factor beta-3	Tgfb3	3.52	Promotes and improves chondrogenesis in MSC populations [46].
*Matrix/cell adhesion/cytoskeleton*			
Collagen, type I, alpha 1	Col1a1	3.03	An extracellular matrix protein that promotes MSC proliferation [47].
Integrin alpha 6	Itga6	−3.25	Also known as CD49f. A cell adhesion molecule that enhances multipotency through direct regulation of OCT4 and SOX2 in human MSC [48].
Melanoma cell adhesion molecule (CD146)	Mcam	(+/−)	Cell adhesion molecule participating in heterotypic intercellular adhesion [49]. Its expression reflects the perivascular origin of the MSC [50].
Matrix metallopeptidase 2	Mmp2	(+/−)	An enzyme that cleaves Type IV collagen of endothelial basement membranes and enhances the migration potential of MSC through the endothelium [51].
Nestin	Nes	(+/−)	An intermediate filament neural stem cell marker whose expression is downregulated during neuronal or glial cell development. Nes+ MSC are quiescent in bone marrow and have high CFU-f activity and trilineage differentiation [52].
Vascular cell adhesion molecule 1 (CD106)	Vcam1	(+/−)	Mediates the attachment of hematopoietic cells [53]. Serves as a marker for MSC with T-cell immunosuppressive activity [54].
Vimentin	Vim	5.05	A major intermediate filament of MSC that plays a positive role in MSC migration [55].
*Immune-related/miscellaneous*			
Frizzled homolog 9 (Drosophila)	Fzd9	(−/+)	A receptor for Wnt-2; functions in Wnt/*β*-catenin signaling [56] that can regulate phase-specific functionality of MSCs [57]. Upregulated in early stages of osteogenic differentiation [58].
Jagged 1	Jag1	22.11	Cell-surface antigen of Notch1; required for regulatory T-cell expansion induced by MSC [59].
Interleukin-6	Il-6	(+/−)	Plays a role in inhibition of lymphocyte apoptosis by MSC [60]. MSC inhibit dendritic cell differentiation through IL-6 [61].
Leukemia inhibitory factor	Lif	(+/−)	Upregulates pluripotency markers in adipose-tissue derived mesenchymal stem cells [62].
Notch gene homolog 1 (Drosophila)	Notch1	(+/−)	Cell surface receptor for Jagged 1; required for Treg-cell expansion induced by MSC [59].
