# Corrigendum to “Mouse Mesenchymal Progenitor Cells Expressing Adipogenic and Osteogenic Transcription Factors Suppress the Macrophage Inflammatory Response”

**DOI:** 10.1155/2019/8428417

**Published:** 2019-04-01

**Authors:** Natalie Fernandez, Heather Renna, Lauren McHugh, Katie Mazalkova, William Crugnola, Jodi F. Evans

**Affiliations:** ^1^Department of Biology, Chemistry and Environmental Studies, Molloy College, 1000 Hempstead Avenue, Rockville Centre, NY 11570, USA; ^2^Biomedical Research Core, Winthrop University Hospital, 101 Mineola Blvd., Mineola, NY 11501, USA; ^3^Stony Brook University School of Medicine, Stony Brook, NY 11794, USA

In the article titled “Mouse Mesenchymal Progenitor Cells Expressing Adipogenic and Osteogenic Transcription Factors Suppress the Macrophage Inflammatory Response” [1], the name of the fourth author was given incorrectly as Mazolkova. The author's name should have been written as Mazalkova. The revised authors' list is shown above.
